# In-Situ Investigation of Copepod Predators of *Ichthyophthirius multifiliis* Theronts from Fish-Farming Pond

**DOI:** 10.3390/microorganisms13010038

**Published:** 2024-12-27

**Authors:** Lijun Wang, Bingwen Xi, Kai Chen, Jun Xie, Liangkun Pan

**Affiliations:** 1 Wuxi Fisheries College, Nanjing Agricultural University, Wuxi 214081, China; 2022113006@stu.njau.edu.cn; 2 Key Laboratory of Aquatic Animal Nutrition and Health, Freshwater Fisheries Research Center, Chinese Academy of Fishery Science, Wuxi 214081, China; chenk@ffrc.cn (K.C.); xiej@ffrc.cn (J.X.); panlk@ffrc.cn (L.P.)

**Keywords:** *Ichthyophthirius multifiliis*, *Cyclops vicinus*, theront, copepods, biocontrol

## Abstract

*Ichthyophthirius multifiliis*, a parasitic ciliate, causes “white spot disease” in freshwater fish and poses a significant threat to global freshwater aquaculture. Eliminating the free-swimming theront stage from the aquaculture environment is a critical measure for controlling *I. multifiliis* infections. The natural predator of *I. multifiliis* theronts in fish-farming ponds were identified using fluorescent dye-labelled live theronts and quantitative PCR; meanwhile, the zooplankton community composition in the positive ponds of *I. multifiliis* detected by quantitative PCR were analyzed by eDNA metabarcoding assay. The results revealed predation on theronts by cyclopoid copepods, including *Cyclops vicinus*, *Thermocyclops taihokuensis*, *Cyclops* sp., *Thermocyclops* sp., *Eucyclops* sp., and *Mesocyclops* sp. from the in-situ predation aquatic ecosystem, and among these copepods, *C. vicinus* was identified as a natural dominant predator of *I. multifiliis*. This study provides a scientific basis for further exploration and utilization of natural predators to enhance sustainable and environmentally friendly control strategies against *I. multifiliis*.

## 1. Introduction

*Ichthyophthirius multifiliis* is a parasitic ciliate, causing white spot disease in freshwater fish globally, and its heavy infection on the gills and skin causes mass mortality in aquaculture [1,2]. The life cycle of *I. multifiliis* is relatively simple, with no intermediate hosts, and includes three functionally distinct stages: the infective theront stage, parasitic trophont stage, and reproductive tomont stage [3]. The infective and free-swimming theronts invade the gills and skin of fish, penetrate the epithelium, and rapidly transform into trophonts, which feed on host tissue and grow up to 0.5–1.0 mm white spots in 7–10 days; the mature trophonts leave host fish and form tomonts, which in turn divides by binary fission and produce numerous theronts [1,4,5]. Due to its low host specificity, *I. multifiliis* infects a wide range of freshwater fish species and poses a significant threat to global freshwater aquaculture [6,7].

Historically, malachite green, mercuric acetate, and their derivatives were used as effective chemical bath treatments against white spot disease in aquaculture, but they were soon banned due to the hazard to environment and consumers [8]. On farms, physical methods such as temperature elevation, ultraviolet irradiation, and water flow are frequently employed to mitigate the occurrence of *I. multifiliis* infection. However, the efficacy of those approaches is limited [9,10,11]. Formalin and copper sulfate are also used to treat this disease, but the inevitable repeated applications have a harmful effect on the water environment and fish health [12,13]. Consequently, there is an urgent need to explore new environmentally friendly control measures for the prevention and treatment of ichthyophthiriasis [14,15,16].

Biological control is considered as a feasible and benign approach to control fish white spot disease [15,17]. Recently, an indoor co-culture experiment revealed that cyclopoid copepods *Thermocyclops taihokuensis*, *Mesocyclops* spp., and *Macrocyclops* sp. *Paracyclopina* sp. could predate the theronts of *I. multifiliis* [17]. The predators may be able to ingest the theronts effectively and thereby reduce infection pressure of *I. multififiliis* in water systems. On fish farms, it has been observed that the eutrophic water body with abundant plankton was less susceptible to the disease outbreaks of *I. multifiliis*. In contrast, the oligotrophic water with few plankton, such as indoor recirculating systems, spring water, or river cage cultures, had a high risk. In the pelagic food web of freshwater ecosystems, the trophic interactions between ciliates and zooplankton have received significant attention, and the predation of ciliates by copepods has been widely investigated [18,19]. For example, the calanid copepod *Calanus sinensis* has been found to exhibit a selective feeding preference for ciliates, with clearance rates significantly higher for ciliates (>20 μm) [20]. The infective theronts of *I. multifiliis*, with a body size of 20–50 μm, is free-swimming in water when seeking a fish host. Therefore, the theronts could be predated upon by the metazooplankton in the aquatic ecosystem. 

In this study, authors tracked theronts labeled with the live-cell fluorescent dye CFSE to identify the predators in fish farming pond. The predators were identified by the morphological characteristic and DNA sequences, while the copepod species diversity in the plankton community was determined by eDNA metabarcoding analyses.

## 2. Materials and Methods

### 2.1. Ichthyophthirius multififiliis Isolation and Fluorescence Labeling 

*I. multififiliis* isolates were collected from infected goldfish (*Carassius auratus*) and maintained in an indoor recirculating water system as described by Cao et al. (2023) [17]. The heavily infected fish were placed in beakers containing aerated tap water and their body surfaces were gently rubbed. Mature trophonts and free-living protomonts were transferred using plastic pipettes to glass culture dishes filled with aerated water and repeatedly washed to remove adhering fish mucus. A final concentration of 1 μM 5-(and 6)-carboxyfluorescein diacetate succinimidyl ester (CFDA-SE) solution (Bestbio, Shanghai, China) was added to the culture dishes, which were then placed in a 23 °C incubator, and kept in darkness for 1 h. The trophonts were separated from the staining solution by 40 µm cell strainers and transferred to new culture dishes containing aerated water. To detect the stability of fluorescence and vitality of theronts, the culture dishes were then kept at 23 °C in darkness for 16–18 h, and examined using a fluorescence stereomicroscope (Nikon SMZ18, Tokyo, Japan). The concentration of theronts suspension was determined and adjusted to 2000 theronts/mL according to the previously described method [21]. The animal study was reviewed and approved by the protocols used on the experimental fish and followed the guidelines of the Institutional Animal Care and Ethics Committee of Nanjing Agricultural University, Nanjing, China (Permit number: SYXK (Su)2011-0036).

### 2.2. Pond Selection and Collection of Water Samples

Twenty-one (21) ponds at the Nanquan experimental station, Freshwater Fisheries Research Center, Chinese Academy of Fishery Sciences, Wuxi, China, were chosen based on fish that had not experienced outbreaks of *I. multifiliis* disease. Pond water (2 L) was collected from the low layers of each pond in triplicate. The samples were immediately filtered through a 40-mesh sieve to remove large particles and transferred to laboratory on ice (0–4 °C). The water samples were immediately vacuum-filtered (5 μm pore size), and the filters were cut into pieces and then stored at −20 °C for further quantitative PCR detection.

### 2.3. In-Situ Predation Experiment

Three net cages (100 mesh, 0.5 × 0.5 × 0.5 m) were placed in ponds detected as *I. multifiliis* positive in Section 2.2, and the pond water temperature was 13–15 °C during the experimental period. In total, 125 L water was collected using a 230 μm mesh plankton net from the same pond and the filtered plankton were added in each cage separately. Meanwhile, one plankton sample set was determined as the control group, and was immediately anesthetized with 75% ethanol (General-reagent^®^, Shanghai, China). About 100,000 fluorescence-labeled theronts were added into the cages, and 3 h later the plankton were recollected using 0.15-mm mesh sieves and immediately anesthetized with 75% ethanol (General-reagent^®^). All samples were then kept on ice and transported to the laboratory. The samples from the experimental group were observed under the fluorescence stereomicroscope (Nikon SMZ18) to collect zooplankton carrying fluorescent signals, which were identified based on morphological characteristics according to Shen (1979) [22].

### 2.4. DNA Extraction and PCR Amplification

Pond water sample DNA were extracted using the E.Z.N.A.^®^ Water DNA Kit (Omega, GA, USA) and detected by quantitative PCR with the *I. multifiliis*-specific primers qIchR and qIchF as described by Cao et al. (2024) [23]. Briefly, qPCR was conducted in a total reaction volume of 20 μL, which included 10 μL of ChamQ SYBR qPCR Master Mix (Vazyme, Nanjing, China), 0.4 μL each of forward and reverse primers (10 μmol/L), 7.2 μL of ddH_2_O, and 2 μL of template DNA. The qPCR reaction program was set as: initial denaturation at 95 °C for 30 s; 40 cycles of 95 °C for 10 s, 60 °C for 30 s for annealing and extension, with signal collection, followed by a melting curve analysis.

Zooplankton from the in situ predation experiment were individually placed in 1.5 mL PE tubes, and 30 μL of Lysis Buffer (Takara, Kyoto, Japan) was added to extract the genomic DNA. Quantitative real-time PCR was performed using the *I. multifiliis*-specific primers qIchR/F to determine whether the zooplankton had ingested theronts, with the reaction system and program as previously described. Additionally, the zooplankton-specific primers ZplankF1/R1 [24] were used to amplify the *CO1* gene for zooplankton species identification. PCR was conducted in a total reaction volume of 25 μL, which included 12.5 μL of 2 × Taq Master Mix (Vazyme), 1.0 μL each of forward and reverse primers, 2 μL of template DNA, and 8.5 μL of ddH_2_O. The PCR amplification conditions were as follows: initial denaturation at 94 °C for 3 min; 36 cycles of 94 °C for 30 s, 58 °C for 30 s, and 72 °C for 15 s; and a final extension at 72 °C for 7 min. PCR products were identified using 2% agarose gel electrophoresis. Visually positive PCR products were selected and sent to Sangon Biotech (Shanghai, China) Co., Ltd. for sequencing. Those amplified by zooplankton-specific primers were sequenced with primers M13F/M14R [24]. The sequences were edited using SeqMan (DNASTAR) [25] and compared against NCBI.

### 2.5. DNA Metabarcoding Assay of Zooplankton Diversity 

Zooplankton were filtered through a 230 μm mesh size plankton net from ponds that were *I. multifiliis* infection positive at a depth of 0.5 m. The samples were immediately rinsed into sampling bottles using pond water and transported to the laboratory. The samples were pre-filtered through a 2.0 mm mesh sieve to remove large particulate debris, followed by vacuum filtration using 5 μm filters to concentrate the zooplankton. The collected filters were stored at −80 °C for high-throughput sequencing.

The zooplankton community in fishponds were investigated using an environmental DNA metabarcoding assay. The DNA extraction, PCR amplification, and high-throughput sequencing of zooplankton samples were processed at a commercial lab (Shanghai BIOZERON Co., Ltd., Shanghai, China). The total genomic DNA for each sample was extracted using a PowerWater DNA isolation kit (Qiagen, San Diego, CA, USA) according to the manufacturer’s instructions. The purity and concentration of DNA was determined using NanoDrop ND-1000 Spectrophotometer (NanoDrop, Boston, MA, USA), and the qualified DNA was amplified with the primers mlCOIintF-XT and jgHCO2198 [26] to obtain the variable regions of mitochondrial *CO1* gene, approximately 313 base pairs. The amplicon libraries of PCR products were sequenced using MiSeq platform (Shanghai BIOZERON Co., Ltd.). After high-throughput sequencing, the raw data were processed to obtain optimized valid sequences. The DADA2 algorithm was employed to calculate ASVs (amplicon sequence variants) and generate representative ASV sequences [27]. These sequences were uploaded to the Silva138.1 database for species matching and taxonomic annotation, resulting in the corresponding ASV abundance table.

### 2.6. Data Statistics and Analyses

Species composition and abundance analyses for plankton diversity were conducted at the species level. For the zooplankton samples collected during the in situ predation experiment co-culture experiments, relative abundance was defined as the proportion of a particular species’ individuals relative to the total individuals within its genus. High-throughput sequencing results were manually curated to exclude non-zooplankton data, and species sequence proportions at each sampling site were calculated to represent relative abundance [28].

## 3. Results

### 3.1. Feasibility of Theronts Labeled with CFDA-SE

**Figure 1:**
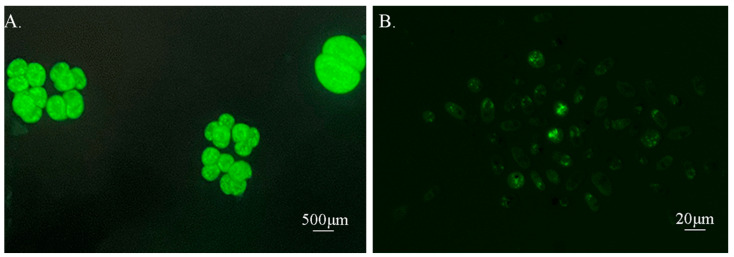
CFDA-SE labeled tomonts and theronts of *I. multifiliis*. ((**A**), tomonts. (**B**), theronts).

The CFDA-SE could effectively label the tomonts and theronts of *I. multifiliis*, which emitted a strong and uniform green fluorescence under a fluorescence stereomicroscope. The development of tomonts were not significantly impacted by the CFDA-SE, while over 90% tomonts were able to divide normally and produced free-swimming theronts after approximately 18 h. These theronts exhibited good motility and emitted significant green fluorescence (Figure 1).

### 3.2. High Covert Infection with I. multifiliis in Ponds

According to the quantitative PCR test of environmental DNA samples, in the twenty-one pond water samples, only six were tested as *I. multifiliis* positive (28.57%). The quantitative PCR Ct values of the six positive samples were between 30.5–35.0, which indicated the abundance of the target gene or *I. multifiliis* cells were low. Based on the relationship between Ct values and theronts concentration established by Cao et al. (2024) [23], theronts in the pond water were 1–10 cells/L. 

### 3.3. In-Situ Natural Predators of Theronts in Pond

**Figure 2:**
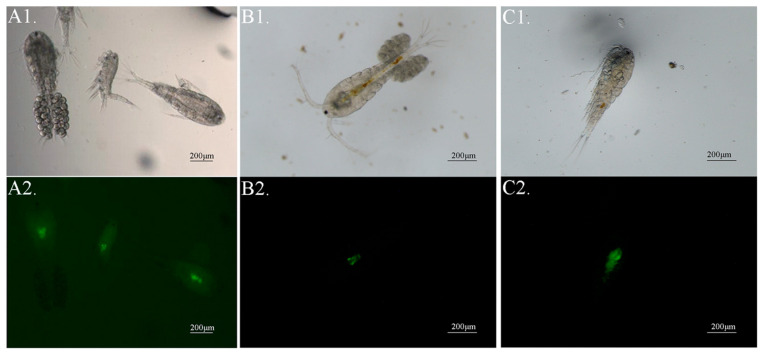
Significant fluorescent signals in the digestive tract of copepods. (**A1**–**C1**) Copepods under bright-field. (**A2**–**C2**), Copepods under dark-field fluorescent light source.

**Figure 3:**
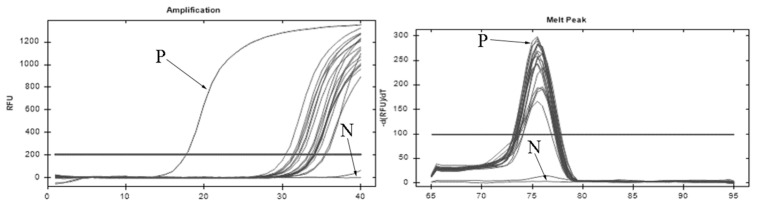
Copepods with CFDA-SE signal were DNA positive of *I. multifiliis*. P, positive control; N, negative control.

The zooplankton in the control group were not detected the CFDA-SE fluorescence signal. In the experiment group, marked signals were observed in the digestive tract of zooplankton, and the fluorescence signal was only detected in mature copepods (Figure 2), although lots of cladocerans and rotifers were observed in the plankton community. The quantitative PCR tests further confirmed that copepods with CFDA-SE signal were DNA positive for *I. multifiliis* (Figure 3). 

The CFDA-SE positive copepods were used to amplify the mitochondrial *CO1* sequences, and 162 individuals were successfully sequenced. Combining the morphological characteristics and *CO1* molecular sequences, six copepod species were identified, all belonging to Cyclopoida. *Cyclops vicinus* was the most dominant predator, accounting for a significant 82.72% of the total predators, followed by *Cyclops* sp. (6.79%), *Thermocyclops taihokuensis* (4.94%), *Eucyclops* sp. (3.70%), *Thermocyclops* sp. (1.23%), and *Mesocyclops* sp. (0.62%) (Table 1). Meanwhile, the percentage of *C*. *vicinus* in NQ1, NQ2, and NQ3 was notably higher than other predatory species. Due to the morphological similarities among zooplankton and limited genetic data, some copepods were only identified to the genus level.

### 3.4. Zooplankton Composition Based on mt CO1 DNA Metabarcoding Assay

**Figure 4:**
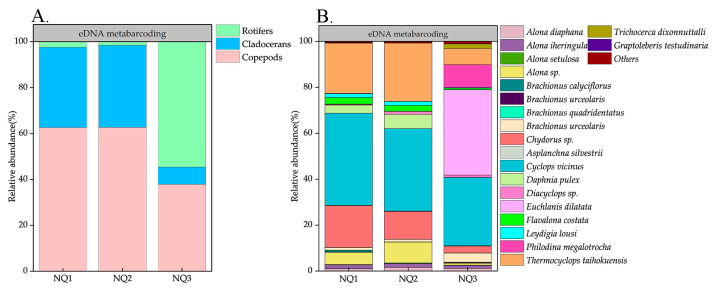
(**A**) relative abundance (%) of copepods, cladocerans, and rotifers and (**B**) relative abundance (%) top 20 of zooplankton at the species level with eDNA metabarcoding in every fish-farming pond.

For eDNA metabarcoding, a total of 273,815 reads were obtained, with the number of assigned reads from each site ranging from 82,707 to 98,946 (mean ± SD: 91,271 ± 8156) in the three *I. multifiliis*-positive ponds. A total of 78 zooplankton from 2 phyla, 6 orders, 22 families, and 39 genera were recorded by eDNA metabarcoding (Table 2 and Table S1). Copepods, cladocerans, and rotifers corresponded to 14, 26, and 38 species, accounting for 17.93%, 33.33%, and 48.72%, respectively (Table 2). Regarding the relative abundance of zooplankton, for eDNA metabarcoding, copepods were the main dominant group, accounting for 55.16% of the total abundance, while cladocerans and rotifers accounted for 26.94% and 17.91%, respectively. At the order level, Cyclopoida (55.07%), Cladocera (26.94%), and Ploima (14.73%) were the main dominant orders. At the species level, the most abundant species was *C*. *vicinus* (35.49%), followed by *T*. *taihokuensis* (18.71%) and *Chydorus* sp. (11.47%) (Figure 4).

For eDNA metabarcoding, 47, 49, and 68 species were obtained in the NQ1, NQ2, and NQ3, respectively (Table 2). In addition, cladocerans were the richest group, accounting for 46.81% and 46.94% of the total species in the NQ1, NQ2, respectively; rotifers were the richest group, accounting for 52.94% in the NQ3 (Table 2). In terms of relative zooplankton abundance for eDNA metabarcoding, copepods were the most dominant group in NQ1 and NQ2, making up 62.59% and 62.69% of the total abundance, respectively, while cladocerans and rotifers accounted for 34.90% and 35.77% and 2.51% and 1.55%, respectively. However, rotifers were the most dominant group in NQ3, making up 54.64% of the total abundance, while cladocerans and copepods accounted for 7.49% and 37.87%, respectively. At the species level, *C*. *vicinus* was the most dominant species in NQ1 and NQ2, accounting for 40.23% and 35.90% of the total abundance, respectively, followed by *T*. *taihokuensis* with 21.96% and 25.39%, *Chydorus* sp. with 18.30% and 12.18%, and *Daphnia pulex* with 3.52% and 6.23%. *Euchlanis dilatata* was the most dominant species in NQ3, accounting for 37.15% of the total abundance, followed by *C*. *vicinus* (29.72%), *Philodina megalotrocha* (9.98%), and *T*. *taihokuensis* (7.08%) (Figure 4).

## 4. Discussion

The ciliate-copepod trophic coupling is well documented from indoor and field investigation, and copepods could significantly impact ciliate population through direct predation [29,30]. The life cycle of *I. multifiliis* involves a free-swimming theront stage in the water, which exhibits morphological and behavioral similarities to ciliates [31]. These similarities may trigger zooplankton to prey on theronts. However, to our knowledge, very few studies have attempted to use zooplankton to control *I. multifiliis* infections in aquaculture. In laboratory conditions, co-cultured zooplankton with fluorescently labeled theronts and observed fluorescent signals in the digestive tracts of copepods were found [17]. Therefore, it is plausible that zooplankton can prey on theronts in wild water bodies. To more accurately reflect the complex interactions between species in natural environments, this study designed in situ predation experiment in fish-farming ponds to verify predation of zooplankton on theronts. We tracked significant fluorescent signals in the digestive tracts of numerous mature copepods, consistent with the findings of Cao et al. (2023) [17]. Additionally, more direct evidence from this study is the positive-*I. multifiliis* nucleic acids in fluorescently marked copepods through quantitative fluorescence assays, indicating their predation on theronts. Thus, this study confirms that in natural aquatic environments, certain copepods can prey on theronts. Furthermore, the predatory role of zooplankton could be an effective approach in *I. multifiliis* control strategies. However, due to the high motility, specialized host-seeking behavior, and short free-living stage of theronts [32,33], whether copepods can effectively prey on them remains an open question. 

Copepods play a crucial ecological regulatory role in ecosystems [34,35] and may offer a potential natural mechanism for controlling *I. multifiliis* infections. The eDNA metabarcoding assay revealed there were 14 copepods, 26 cladocerans, and 38 rotifers identified from the three *I. multifiliis*-positive ponds. The relative abundance analysis of zooplankton revealed that copepods accounted for 55.16% of the total zooplankton, while cladocerans and rotifers represented 41.67%. However, most zooplankton that ingested the theronts were identified as copepods, with few cladocerans or rotifers observed in the in situ predation experiment. This finding suggests that copepods may be the dominant group preying on theronts. Due to this study being conducted in only three fish ponds, there may be more predators could be found from different commercial aquaculture systems. In ecosystems, most rotifers and cladocerans feed by swimming and filtering particles from the water, whereas copepods employ active sensing and ambush strategies to capture larger prey, often exhibiting selective preference for ciliates [17,36]. Additionally, quantitative PCR showed that the gene abundance of *I. multifiliis* was at a relatively low level, and no ichthyophthiriasis outbreaks were observed before or after the in situ predation experiments. eDNA metabarcoding assay further showed that copepods were the dominant group in NQ1 and NQ2, comprising over 60%. Although rotifers were the dominant group in NQ3, copepods still accounted for over 30%. This raises the question of whether the high abundance of copepods in these ponds is related to the suppression of *I. multifiliis*. In previous studies, Cao et al. (2023) found that the presence of zooplankton significantly reduced the intensity of *I. multifiliis* infections in goldfish [17]. Similarly, Kim Hue et al. (2019) reported that *Acanthocyclops robustus* (Copepoda: Cyclopoida) effectively eliminated pest ciliates (*Sterkiella* sp.) in microalgae cultures, reducing ciliate contamination in algal systems [37]. Therefore, we hypothesize that rich copepods in the water may contribute positively to reducing the abundance of *I. multifiliis*, thereby partially inhibiting its spread and the associated infection risk.

This study identified six zooplankton species capable of preying on theronts, all belonging to the order Cyclopoida based on morphological characteristics and *CO1* molecular sequences. Among these copepods, *C. vicinus* was the most dominant species, accounting for 82.7% of the predatory zooplankton, whereas *T. taihokuensis* comprised only 4.8%. Conversely, Cao et al. (2023) identified seven copepod species capable of preying on theronts in a laboratory co-culture predation experiment, with *T. taihokuensis* being particularly prominent, representing 62.5% of predators identified [17]. This discrepancy may be attributed to different season of sampling. Seasonal changes significantly influence the community of copepods in aquatic ecosystems, resulting in distinct seasonal fluctuations in their density and biomass [38]. Research indicates that *C. vicinus* is most active between December and May, thriving in cooler conditions and serving as a dominant species during winter and spring [39,40,41]. In contrast, *T. taihokuensis* exhibits peak activity from June to November, making it a dominant species in summer and autumn [38,42]. The sampling in this study was conducted in spring (March), and eDNA analyses revealed that *C. vicinus* exhibited the highest relative abundance among copepods, showing its ecological dominance during this period. Suitability of environmental conditions likely conferred competitive advantages and enhanced predation efficiency of *C. vicinus*, enabling it to dominate theront predation. However, of the theront predators from fishponds identified in this study and Cao et al. (2023) [17], all were investigated at different times and over short periods. Further studies to reveal the seasonal dynamics of copepod community and the dominant theront predators from fishponds in the long term are needed. 

Recently, biological control has been widely implemented in the prevention and management of aquaculture pests and diseases [43,44]. This study identified a strong spatiotemporal correlation between *C. vicinus* and *I. multifiliis*, suggesting that *C. vicinus* holds significant potential for controlling *I. multifiliis*. Based on these findings, it is recommended to prioritize the development of pond ecosystem-based strategies for conserving *C. vicinus* populations or establishing large-scale monoculture systems for *C. vicinus*. Such approaches could enhance its pest-control efficacy and promote the advancement and application of sustainable, green control technologies for managing *I. multifiliis*.

## Figures and Tables

**Table 1:** Copepods of theronts of *I. multifiliis* discovered in this study.

Source	Species	Numbers	Genbank Similar Species	
			Species (Acc. No.)	Sequence Similarity
NQ1	*Cyclops vicinus*	45	*C. vicinus* (LC604938)	99.05~100%
	*Thermocyclops taihokuensis*	3	*T. taihokuensis* (LC215456)	99.08~99.09%
	*Cyclops* sp.	4	*C. vicinus* (LC604938)	96.36%
	*Thermocyclops* sp.	2	*T. taihokuensis* (LC215458)	98.02~98.12%
NQ2	*C. vicinus*	47	*C. vicinus* (LC604938)	99.05~100%
	*T. taihokuensis*	5	*T. taihokuensis* (LC215456)	99.09~99.85%
	*Mesocyclops* sp.	1	*Mesocyclops* sp. (KJ020568)	96.94%
	*Cyclops* sp.	6	*Cyclops* sp. (LC215454)	84.43~98.49%
	*Eucyclops* sp.	2	*Eucyclops* sp. (KJ020567)	98.94~100%
NQ3	*C. vicinus*	42	*C. vicinus* (LC604938)	99.05~100%
	*Eucyclops* sp.	4	*Eucyclops* sp. (KJ020567)	98.94~100%
	*Cyclops* sp.	1	*Cyclops* sp. (LC215454)	84.83%

**Table 2:** The number of species identified using eDNA metabarcoding.

Phylum	Order				Family				Genus				Species		
	NQ1	NQ2	NQ3		NQ1	NQ2	NQ3		NQ1	NQ2	NQ3		NQ1	NQ2	NQ3
Arthropoda	3	3	3		7	8	10		18	21	21		32	35	34
Rotifera	3	3	3		9	7	11		8	4	15		15	14	34
Total	6				22				39				78		

## Data Availability

The authors confirm that the data supporting the findings of this study are available within the manuscript and table. The datasets presented in this study can be found in online repositories. The names of the repository/repositories and accession number can be found below: https://www.ncbi.nlm.nih.gov/, PRJNA1194289 (accessed on 6 December 2024).

## Supplementary Materials

The following supporting information can be downloaded at: https://www.mdpi.com/article/10.3390/microorganisms13010038/s1, Table S1: The zooplankton community in fishponds for eDNA metabarcoding assay.
